# Mitochondrial genetic variation reveals phylogeographic structure and cryptic diversity in *Trioza erytreae*

**DOI:** 10.1038/s41598-020-65880-7

**Published:** 2020-06-01

**Authors:** Inusa Ajene, Gerhard Pietersen, Barbara van Asch

**Affiliations:** 1 Department of Genetics, Stellenbosch University, Private Bag X1, Matieland, 7602 South Africa; 2 Department of Crop Protection, Ahmadu Bello University, Samaru 810001 Zaria, Nigeria; 3 International Center of Insect Physiology and Ecology, P.O. Box 30772, Nairobi, Kenya

**Keywords:** Ecology, Evolution, Genetics, Plant sciences, Ecology

## Abstract

*Trioza erytreae* is the main vector for ‘Candidatus Liberibacter africanus’, the causative agent of African Citrus Greening disease. The insect is widespread in Africa, and has recently disseminated to Southwestern Europe. This study aimed at generating reference mitogenome sequences for *T. erytreae*, as a background for future genetic diversity surveys. Complete mitochondrial sequences of three specimens collected in Ethiopia, Uganda and South Africa were recovered using Ion Torrent technology. The mitogenomes of *T. erytreae* from Uganda and Ethiopia were highly similar, and distinct from that found in South Africa. The phylogeographic structure of *T. erytreae* was assessed using genetic clustering and pairwise distances, based on a dataset of public *COI* sequences recorded as *T. erytreae*. The dataset revealed ten haplotypes with strong phylogeographic structure in Africa and Europe. Three haplotypes found in Kenya on *Clausena anisata* belonged to pairs separated by distances as high as 11.2%, and were basal to all other sequences. These results indicate that not all sequences identified as *T. erytreae* belong to the same species, and that some degree of specificity with different plant hosts is likely to exist. This study provides new baseline information on the diversity of *T. erytreae*, with potential implications for the epidemiology of African Citrus Greening disease.

## INTRODUCTION

Citrus is one of the most important fruit crops globally, with an average annual production of over 73 million metric tons (MT)^1^. Numerous citrus species are commercially cultivated worldwide, including sweet orange (*Citrus sinensis* Osbeck), lemon (*Citrus limon* [L.] Burm. f.), lime (*Citrus aurantifolia* [Cristm.] Swingle), grapefruit (*Citrus paradisi* Macfad), and mandarin (*Citrus reticulata* Blanco). Sweet orange is the most popular citrus, and accounts for more than half of total world production^1^.

Currently, the leading citrus producers in Africa are Egypt and South Africa, with an average of 1.4 million MT of oranges per year in South Africa^1^. In spite of high market demand and great agricultural potential for citrus in Africa, production has been declining^2,3^. For example, the total yield of oranges has gradually decreased from 205,895 hg/ha in 2014 to 198,710 hg/ha in 2017, as well as the total yield of lemons and limes, from 148,431 hg/ha to 135,435 hg/ha, in the same period ^1^. Numerous biological factors constrain citrus production in Africa, including important insect pests (aphids, whiteflies and triozids), and viral and bacterial diseases (Citrus Tristeza and Citrus Greening Disease).

The African citrus triozid, *Trioza erytreae* (Del Guercio) (Hemiptera: Triozidae), is one of the most damaging citrus pests in Africa^2,4^. *Trioza erytreae* was first recorded in 1929 in South Africa^5^, and it is widely distributed on the continent. The insect has been reported in 19 out of the 54 African countries: Angola, Kenya, Ethiopia, Eritrea, Madagascar, Malawi, Mauritius, La Réunion, South Africa, Sudan, Swaziland, St. Helen, Tanzania, Uganda, Zambia, Democratic Republic of the Congo, Rwanda, Comoros, and Cameroon^6^. Outside Africa, *T. erytreae* was first detected in Madeira (Portugal) in 1994^7^, and it has now spread to the southwest of the Iberian Peninsula through northern Spain and Portugal^8^.

*Trioza erytreae* causes damage to citrus plants by larval feeding, which results in notching and curling of the leaves. In addition, the production of honeydew by the insect promotes the growth of sooty mould on the plant, decreasing its productivity. Although the negative impact of larval feeding is significant, the most injurious activity of the insect results from its role as the vector for ‘Candidatus Liberibacter africanus’ (Laf), a phloem-limited bacterium responsible for the African Citrus Greening disease (ACG)^9^. In Eastern and Southern Africa, ACG is reported to have high impact on citrus production, especially in the highlands, causing yield losses of 25% to 100%^10,11^. While *T. erytreae* is the primary vector for Laf^12^, the insect is also able to transmit another citrus pathogen – ‘Candidatus Liberibacter asiaticus’ (Las) - under experimental conditions^13^. Las is associated with the severe Huanglongbing (HLB) disease, normally transmitted by *Diaphorina citri* in Asia, and North and South America^14–16^. In Africa, Las was reported for the first time in Ethiopia in 2009, in the absence of *D. citri*, its primary vector^17^. Recent reports of *T. erytreae* field populations carrying Las in the Ethiopian highlands, in the absence of *D. citri*, support the possibility that the insect is able to transmit the pathogen under natural conditions. Las was also reported on citrus in Uganda^18^ and Tanzania^19^, but this finding was due to a misidentification, and the bacteria detected were most likely ‘Candidatus Liberibacter africanus spp. clausenae’ (LafCl)^20^. Therefore, Las has not yet been confirmed in Uganda or Tanzania. LafCl has only been detected on *Clausena anisata*, an African member of the family Rutaceae^21^, and has not been found on citrus in South Africa^22^. The confirmed presence of Las in Africa, along with the widespread occurrence of *T. erytreae* and its ability to transmit the pathogen, contribute to a scenario of significant threat to citrus production on the continent.

As the control of *T. erytreae* is directly linked to the management of ACG, understanding the bioecology of the triozid is relevant for its management and control^23,24^. The genetic diversity and phylogeographic structure of *T. erytreae* are largely unknown, except for a study which focused on the generation of DNA barcodes for species identification in the genus *Trioza*^25^, and a recent work aimed at testing the host range of *Tamarixia dryi*, a parasitoid wasp associated with *T. erytreae* in South Africa^26^.

The objective of the present study was to establish a background for the assessment of the genetic diversity and phylogeography of *T. erytreae* in Africa, as the species is present in a wide range of bioclimatic regions and different plant hosts, across large geographical distances. For that purpose, we sequenced and compared the complete mitogenomes of three individuals collected in Ethiopia, South Africa and Uganda. The phylogenetic position of *T. erytreae* within the family Triozidae was recovered in the context of publicly available mitogenome sequences. The intraspecific haplotype structure and the genetic diversity of *T. erytreae* were assessed using a compilation of publicly available *COI* sequences.

## Results and Discussion

The complete mitochondrial genomes of three adult specimens of *T. erytreae* collected from citrus plants in Ethiopia (TE-ETH), Uganda (TE-UG) and South Africa (TE-SA) were sequenced with three objectives: 1) to generate reference mitogenomes for future studies, 2) to gain insights into the phylogeographic structure of the species in Africa, and 3) to assess the phylogenetic position of the new mitogenomes within the family Triozidae, using publicly available complete sequences.

### Mitogenomics

The Ion Torrent run resulted in an average of 22.5 million reads for each sample with average read length of 175 bp. The *de novo* assembly generated a contig with 133,872 reads for TE-ETH, a contig with 135,931 reads for TE-UG, and a contig with 31,073 reads for TE-SA. The average sequence coverage was 2,861x for TE-ETH, 2,651x for TE-UG, and 37,460x for TE-SA. A complete mitogenome sequence for *T. erytreae* was available on Genbank (NC_038142.1), and could potentially be used for the mapping and assembly of the new NGS data. However, the sequence contained an unusual, non-annotated 1,689 bp region between *ND2* and tRNA^Trp^, additionally to the annotated AT-rich region (control region). Animal mitogenomes are compact, and do not generally contain large non-coding intergenic regions apart from the control region. Therefore, the new mitogenomes were recovered using *de novo* assembly and reference-based assembly. The mitogenomes recovered using the *de novo* assembly did not have the unannotated 1,689 bp region between *ND2* and tRNA^Trp^ present in NC_038142.1, the only mitogenome available on Genbank for *T. erytreae*. The reference-based mapping using NC_038142.1, after the excision of the unannotated region, resulted in the mapping of 281,007 reads for TE-ETH, 259,315 reads for TE-UG, and 3.6 million reads for TE-SA. The reference based assembly resulted in an average coverage of 3,062x for TE-ETH, 2,835x for TE-UG, and 39,887x for TE-SA. The *de novo* and the reference-based assembly methods resulted in identical sequences for each mitogenome, indicating that the sequence NC_038142.1 should be revised, as the 1,689 bp region between *ND2* and tRNA^Trp^ most probably represents a sequencing or assembly artefact.

**Figure 1:**
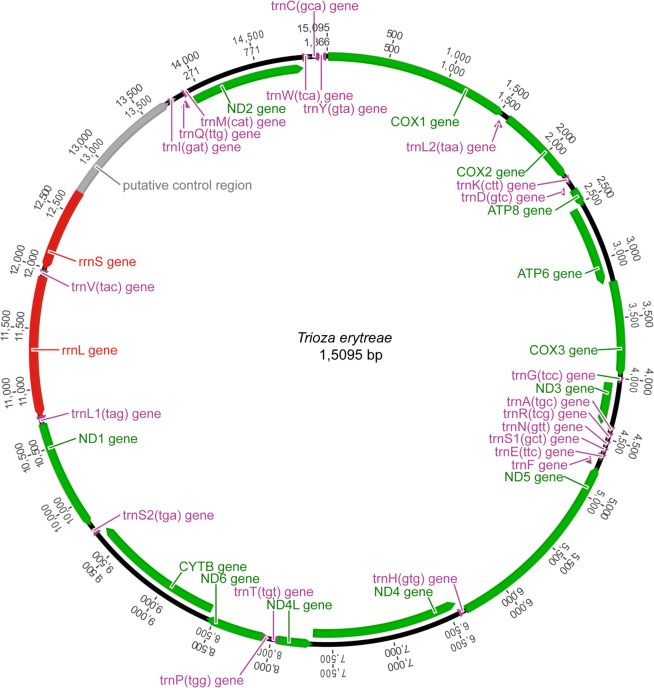
Mitogenome organization of *Trioza erytreae*. Circular map of the mitochondrial genome of *Trioza erytreae*. Protein-coding, transfer RNAs and ribosomal genes are shown with standard abbreviations. The arrows indicate the direction of the genes.

The main features were identical in the three mitogenomes, with the typical mitochondrial complement of 13 protein-coding genes (PCGs), 22 transfer RNA (tRNA) genes, two ribosomal RNA (rRNA) genes, and an AT-rich non-coding region generally assumed to contain the control for transcription and replication (Fig. 1). Gene order was conserved in all species included in the phylogenetic reconstruction (Table S1), and identical to the hypothetical ancestral mitogenome organisation in insects. The average size of the complete sequences (15,087 bp) was similar to the average size of the complete mitogenomes of other triozids (15,028 bp). Twenty-three genes were located on the majority (J) strand, and 14 genes were located on the minority (N) strand.

The three complete mitogenomes had the high A + T content characteristic of insects, with values higher than 79.9% (complete sequence) in all genes (Table S2). The average A + T content of the AT-rich region (88.6%) was higher than the average for the complete sequences (80.0%), the combined tRNAs (79.0%) and the two rRNAs (79.8%). All PCGs had negative GC-skews, except for *COI*. Five PCGS (*ATP8*, *ND4*, *ND4L*, *ND1* and *ND5*) had positive AT-skews, while eight PCGs (*COII*, *ATP6*, *COIII*, *ND3*, *ND6*, *CYTB* and *ND2*) had negative AT-skews (Table S2). The three mitogenomes were equally compact, and had 13 short gene overlaps mostly involving tRNAs. Gene overlaps ranged between 1 and 13 bp, with the largest between tRNA^Glu^ and tRNA^Phe^. Intergenic regions were found at 15 locations, and represented a total average of 78 bp, the longest of which was between tRNA^Ser2^ and *ND1* (25 bp). The largest non-coding region (average = 1,054 bp) was located between the 12S rRNA and the I-Q-M tRNA cluster, and was annotated as the putative control region. The large ribosomal RNA gene (16s rRNA; 1,146 bp) was located between tRNA^Leu1^ and tRNA^Val^, and the small ribosomal RNA gene (12s rRNA; 685 bp) was located between tRNA^Val^ and the AT-rich region. The total complement of 22 tRNAs was identified with ARWEN software, and the sizes of the genes varied between 52 bp (tRNA^His^) and 69 bp (tRNA^Lys^ and tRNA^Gln^). The average combined length of the 13 PCGs was 10,822 bp, representing 71.7% of the total mitogenomes, similarly to other triozids (average 10,801 bp) (Table 1). The largest PCG was *ND5* (1,618 bp), and the smallest was *ATP8* (153 bp), also in line with the other triozids. The PCGs had an average A + T content of 79.4%, ranging from 75.6% in *COI* to 85.7% in *ATP8*. The most frequent start codon was ATG, and all genes were terminated by TNN codons (TAA, TTA, TAG and T) (Fig. S1).

**Table 1:** Mitogenome features of the complete sequence of three *Trioza erytreae* specimens collected in Ethiopia, Uganda and South Africa.

Region	Code	Strand	Anticodon	Start	Stop	*Trioza erytreae* Ethiopia			*Trioza erytreae* Uganda			*Trioza erytreae* South Africa		
						Coordinates	Size (bp)	IGN	Coordinates	Size (bp)	IGN	Coordinates	Size (bp)	IGN
COI	—	J	—	ATG	TAA	1–1533	1533	—	1–1533	1533	—	1–1533	1533	—
tRNA^Leu2^	L2	J	TTA	—	—	1530–1590	61	−4	1530–1590	61	−4	1530–1590	61	−4
COII	—	J	—	ATT	T–	1590–2253	664	−1	1590–2253	664	−1	1590–2253	664	−1
tRNA^Lys^	K	J	AAG	—	—	2255–2323	69	1	2255–2323	69	1	2255–2323	69	1
tRNA^Asp^	D	J	GAC	—	—	2323–2381	59	−1	2323–2381	59	−1	2323–2381	59	−1
ATP8	—	J	—	ATT	TAA	2381–2533	153	−1	2381–2533	153	−1	2381–2533	153	−1
ATP6	—	J	—	ATG	TAA	2527–3201	675	−7	2527–3201	675	−7	2527–3201	675	−7
COIII	—	J	—	ATG	TAA	3201–3983	783	−1	3201–3983	783	−1	3201–3983	783	−1
tRNA^Gly^	G	J	GGA	—	—	3987–4045	59	3	3987–4045	59	3	3987–4045	59	3
ND3	-	J	—	ATT	TAG	4045–4395	351	−1	4045–4395	351	−1	4045–4395	351	−1
tRNA^Ala^	A	J	GCA	—	—	4395–4454	60	−1	4395–4454	60	−1	4395–4454	60	−1
tRNA^Arg^	R	J	CGA	—	—	4455–4516	62	—	4455–4516	62	—	4455–4516	62	—
tRNA^Asn^	N	J	AAC	—	—	4517–4580	64	—	4517–4580	64	—	4517–4580	64	—
tRNA^Ser1^	S1	J	AGC	—	—	4581–4635	55	—	4581–4635	55	—	4581–4636	56	—
tRNA^Glu^	E	J	GAA	—	—	4638–4696	59	2	4638–4696	59	2	4639–4697	59	3
tRNA^Phe^	F	N	GAA	—	—	4684–4744	61	−13	4684–4744	61	−13	4685–4745	61	−13
ND5	—	N	—	ATG	T–	4748–6365	1618	3	4748–6365	1618	3	4749–6366	1618	3
tRNA^His^	H	N	CAC	—	—	6374–6425	52	8	6374–6425	52	8	6375–6426	52	8
ND4	—	N	—	ATA	TTA	6423–7667	1245	−3	6424–7668	1245	−3	6424–7668	1245	−3
ND4L	—	N	—	CAA	TAA	7661–7949	288	−7	7661–7949	288	−7	7662–7949	288	−7
tRNA^Thr^	T	J	ACA	—	—	7951–8012	62	2	7951–8012	62	2	7952–8013	62	2
tRNA^Pro^	P	N	CCA	—	—	8013–8078	66	—	8013–8078	66	—	8014–8079	66	—
ND6	—	J	—	ATT	TAA	8080–8559	480	1	8080–8559	480	1	8081–8560	480	1
CYTB	—	J	—	ATG	TAA	8553–9695	1143	−7	8553–9695	1143	−7	8554–9696	1143	−7
tRNA^Ser2^	S2	J	TCA	—	—	9705–9765	61	9	9705–9765	61	9	9706–9766	61	9
ND1	—	N	—	ATA	TTA	9791–10708	918	25	9787–10709	918	25	9792–10709	918	25
tRNA^Leu1^	L1	N	CTA	—	—	10710–10774	65	1	10710–10774	65	1	10711–10775	65	1
16 s rRNA	—	N	—	—	—	10775–11920	1146	—	10775–11920	1146	—	10776–11923	1148	—
tRNA^Val^	V	N	GTA	—	—	11927–11987	61	6	11927–11987	61	6	11930–11990	61	6
12 s rRNA	—	N	—	—	—	11994–12678	685	6	11994–12678	685	6	11997–12681	685	6
AT-rich region	—	—	—	—	—	12679–13729	1051	—	12679–13729	1051	—	12682–13733	1052	—
tRNA^Ile^	I	J	ATC	—	—	13732–13794	63	2	13732–13794	63	2	13736–13798	63	2
tRNA^Gln^	Q	N	CAA	—	—	13792–13860	69	−3	13792–13860	69	−3	13796–13864	69	−3
tRNA^Met^	M	J	ATG	—	—	13862–13925	64	1	13862–13925	64	1	13866–13929	64	1
ND2	—	J	—	ATG	TAA	13925–14893	969	−1	13925–14889	969	−1	13929–14897	969	−1
tRNA^Trp^	W	J	TGA	—	—	14893–14959	67	—	14893–14959	67	—	14897–14962	66	−1
tRNA^Cys^	C	N	TGC	—	—	14961–15023	63	1	14961–15023	63	1	14964–15026	63	1
tRNA^Tyr^	Y	N	TAC	—	—	15024–15086	63	—	15024–15086	63	—	15027–15089	63	—

J – majority strand; N – minority strand. IGN – intergenic regions, with negative values representing overlapping regions.

**Figure 2:**
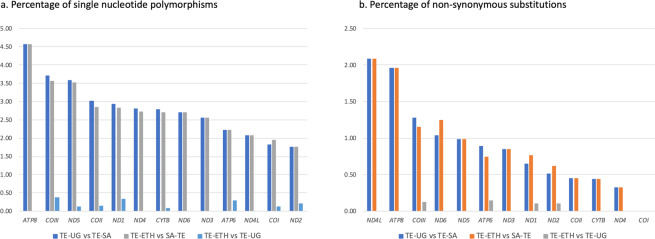
Mitogenome comparisons. Comparison of the mitochondrial sequences of three *Trioza erytreae* specimens collected in Ethiopia (TE-ETH), Uganda (TE-UG) and South Africa (TE-SA), based on the total complement of 13 protein-coding genes. The number of differences is given as (**A**) percentage of single nucleotide polymorphisms, and (**B**) percentage of non-synonymous substitutions, relatively to the size of each gene.

Nucleotide pairwise comparisons showed high similarity between the mitogenomes of the specimens from Ethiopia and Uganda, with only 16 single nucleotide polymorphisms (SNPs) found across the 13 PCGs. All SNPs were transitions, four of which resulted in amino acid changes (Table S3). In PCGs, the largest number of nucleotide differences between TE-ETH and TE-UG was found in *COIII* and *ND1*, with three SNPs in each of the genes. The sequences of *ND6*, *ND3*, *ATP8* and *ND4L* were identical in TE-ETH and TE-UG. In contrast, an average of 294 SNPs was found between TE-SA/TE-ETH and TE-SA/TE-UG, considering only the PCGs. These SNPs represented an average of 261 transitions and 33 transversions, of which 67 resulted in amino acid changes. The percentage of nucleotide differences between TE-SA and the other two mitogenomes was highest in *ATP8* (4.58%), and the percentage of amino acid substitutions was highest in *ND4L* (2.08%) (Fig. 2). Comparison of tRNA sequences showed 16 SNPs between TE-SA and the other two mitogenomes in 10 genes (tRNA^Asp^, tRNA^Arg^, tRNA^Cys^, tRNA^Glu^, tRNA^Gly^, tRNA^Leu2^, tRNA^Pro^, tRNA^Ser1^, tRNA^Ser2^, tRNA^Thr^). In contrast, only one SNP was found between the tRNAs of TE-ETH and TE-UG (tRNA^Trp^).

### Phylogeny of the family Triozidae

**Figure 3:**
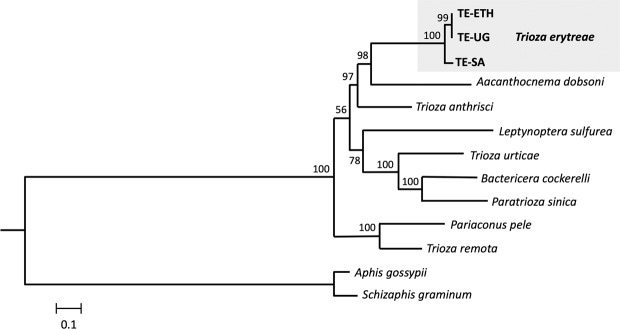
Mitochondrial phylogeny of Triozidae. Maximum likelihood tree representing the phylogenetic relationships within the family Triozidae, using the complete complement of the 13 mitochondrial protein-coding genes. *Aphis gossypii* and *Schizaphis graminum* (Aphididae) were used as outgroups. Values represent nodal support calculated from 1,000 bootstrap replicates. The length of the branches is proportional to the number of substitutions per site.

The phylogenetic position of *T. erytreae* within Triozidae was recovered in a ML tree, using eight other complete mitogenomes publicly available, and two Aphididae as outgroups (Fig. 3). The new *T. erytreae* sequences formed a monophyletic cluster, in accordance with their low level of divergence. The topology of the mitochondrial tree was not fully congruent with that presented in a recent reconstruction of the superfamily Psylloidea, which employed a combination of mitochondrial and nuclear sequence data across a large number of species^27^. However, some common features were recovered: *T. erytreae* and *Aacanthocnema dobsoni* were closely related, in accordance with their classification in group D by Percy *et al*. 2018^27^, as well *Trioza urticae*, *Bactericera cockerelli* and *Paratrioza sinica* in group M, and *Pariaconus pele* and *Trioza remota* in group A. The differences consisted in the position of *Trioza anthrisci* (group A), which was not recovered as closely related to *P. pele* and *T. remota* (group A), and the relative order of the species clusters representing groups. For example, the cluster formed by *T. urticae*, *B. cockerelli* and *P. sinica* (group M), was not recovered as basal to the other taxa. The recovery of deeper phylogenetic nodes is a well-known limitation of mitochondrial-based phylogenies, and the inclusion of nuclear sequence data is often necessary for the assessment of higher divergences^28^. Therefore, these results should be interpreted conservatively.

### Intraspecific diversity of *Trioza erytreae*

The intraspecific genetic diversity of *T. erytreae* was assessed using a dataset of *COI* barcoding sequences identified as *T. erytreae* collected in Africa and Europe in two previous studies^25,26^ (Table S4). The final *COI* dataset (n = 89) included the three sequences generated in the present study, and was strongly biased towards Kenya, which represented 75.2% of all sequences (n = 66), followed by South Africa (14.6%; n = 13). Europe was represented by specimens collected in Portugal and Spain (n = 7), the only countries where *T. erytreae* has been found outside Africa. The *COI* dataset was analysed using a MJ network, a NJ tree, and estimates of genetic divergence (p-distances).

**Figure 4:**
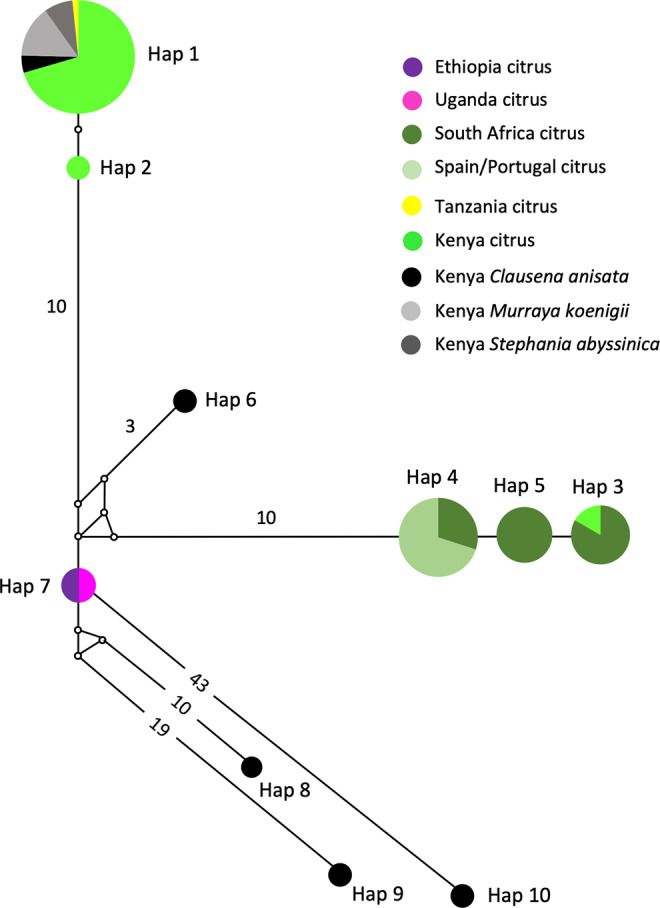
Network of *Trioza erytreae* haplotypes. Median-joining network of cytochrome c oxidase subunit 1 (*COI*) gene (607 bp) of *Trioza erytreae* (n = 89), showing the relationships between haplotypes according to geographic origin and plant host. The size of the circles is proportional to the number of individuals sharing the same haplotype. The length of the branches is proportional to the number of nucleotide substitutions between haplotypes. Numbers along branches indicate the number of substitutions between haplotypes.

The MJ network revealed phylogeographic structuring of *T. erytreae* haplotypes, and their association with the different plant hosts (Fig. 4). Branch I was formed by two closely related haplotypes (Hap1 and Hap2), and included the majority of specimens found on citrus, some specimens found on *Clausena anisata*, *Murraya koenigii* and *Stephania abyssinica* in Kenya, and the single specimen from Tanzania found on citrus. Branch II (Hap3, Hap4 and Hap5) included all specimens collected from citrus plants in Portugal, Spain and South Africa, and one specimen collected in Kenya, also found on citrus. The specimens collected in Portugal and Spain had a single haplotype (Hap4) which was shared with South Africa, and closely related to Hap5 (South Africa) and Hap3 (shared between South Africa and Kenya). This haplotype similarity suggests that *T. erytreae* found in Europe most likely originated from South Africa, although the possibility of a Kenyan origin cannot be excluded. Branch III (Hap6) included a single specimen found in Kenya on *C. anisata*, and Branch IV represented the haplotype found in Ethiopia and Uganda on citrus. Branches V, VI and VII (Hap8, Hap9 and Hap10) represented the highly diverged haplotypes found in Kenya on *C. anisata*.

**Figure 5:**
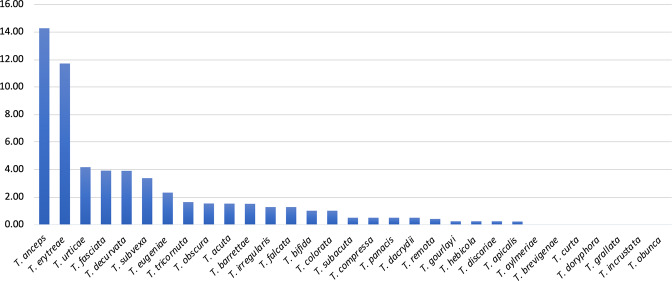
Genetic divergence within *Trioza* species. Intra-specific pairwise distances (K2P) in 31 species of the genus *Trioza* (Triozidae), based on cytochrome c oxidase 1 (*COI*) sequences.

The p-distances among all pairs of haplotypes represented in the network (Hap1 to Hap10) allowed for further inferences on the genetic diversity of *T. erytreae* (Table 2). The high p-distances between Hap8, Hap9 and Hap10 and other sequences, presented in the MJ network in the previous section, was confirmed. Hap10 was the most diverged haplotype, with a p-distance from all other haplotypes higher than 7.65%, followed by Hap9 which diverged from all other haplotypes by more than 5.24%. These values suggest that Hap9 and Hap10 represent two species distinct from all other haplotypes. Hap8 had a different pattern of p-distances relatively to the other haplotypes, as it diverged from Hap1, Hap2, Hap3 and Hap4 by more than 5.06%, suggesting that it represents a distinct species, but had a lower divergence from Hap6 (3.63%, Kenya) and Hap7 (2.82%, Ethiopia and Uganda). Overall, the high divergence among some of the *COI* sequences displayed in the MJ network and the p-distances among the haplotypes indicated that not all specimens morphologically identified as *T. erytreae* in the publicly available dataset belong to the same species. This pattern of high intra-specific divergence found in *T. erytreae* (maximum p-distance = 11.71%) was not observed in the majority of the other 30 Trioza species analysed, for which the average maximum intraspecific p-distance was 1.11%. The exceptions were *Trioza urticae* (4.18%), and *Trioza anceps* (14.27%), which may represent cases of accidental misidentification or cryptic diversity (Fig. 5; Table S5).

**Table 2:** Genetic divergence among *Trioza erytreae* haplotypes, calculated as percentage of pairwise distances under the Kimura 2-parameter model.

	Hap 1	Hap 2	Hap 3	Hap 4	Hap 5	Hap 6	Hap 7	Hap 8	Hap 9	Hap 10
Hap 1	—									
Hap 2	0.32	—								
Hap 3	2.92	2.92	—							
Hap 4	2.59	2.59	0.64	—						
Hap 5	2.76	2.76	0.48	0.16	—					
Hap 6	2.69	2.69	3.03	2.52	2.69	—				
Hap 7	2.59	2.59	2.75	2.06	2.23	1.00	—			
Hap 8	5.24	5.24	5.42	4.89	5.06	3.63	2.82	—		
Hap 9	7.20	7.20	7.56	7.01	7.19	6.18	5.24	5.37	—	
Hap 10	9.37	9.37	10.15	9.56	9.76	8.92	7.65	8.79	11.20	—

**Figure 6:**
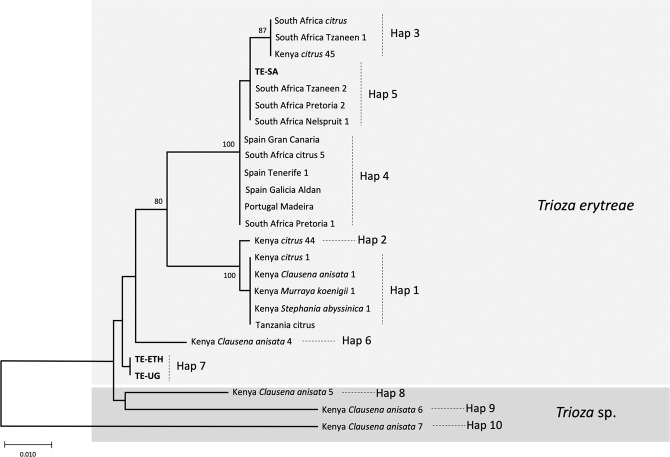
NJ tree of *Trioza erytreae* haplotypes. Neighbour-joining tree representing the relationships among haplotypes of *Trioza erytreae* collected from citrus and other plant hosts in Africa and Europe. The tree was constructed using a 571-bp alignment of cytochrome c oxidase subunit 1 (*COI*) sequences. Nodal support was calculated using 1,000 bootstrap replicates. The length of the branches is proportional to the number of substitutions per site.

Haplotypes are designated according to the median-joining network (Fig. 4), and the branches in the neighbour-joining tree (Fig. 6).

The relationships among *COI* sequences of *T. erytreae* were also represented using an NJ tree (Fig. 6), which recovered the pattern of genetic divergence among haplotypes evidenced in the MJ network. The most basal sequences in the NJ tree were Hap8, Hap9 and Hap10 - all represented by specimens found in Kenya on *C. anisata* - in accordance with the high p-distances among these haplotypes and the other sequences. The bootstrap support for these basal nodes was low, as typically occurs in trees constructed using short *COI* sequences among distantly related taxa^29^.

### African citrus triozids may represent a species complex

In face of the overall results, a hypothesis explaining the genetic diversity of *T. erytreae* is presented in the NJ tree, and proposes that Hap8, Hap9 and Hap10 constitute three cryptic *Trioza* sp. distinct from *T. erytreae*, which is represented by the remaining haplotypes. However, this hypothesis is based on the analyses of a low number of sequences representing a short region of *COI*, a genetic marker that should not be used for species delimitation^30^. Therefore, our proposal is probably an oversimplification of the complex phylogenetic relationships among African citrus triozids, and additional genetic, morphological and ecological data will be necessary to draw definite conclusions. Nevertheless, these results indicate cryptic diversity in *T. erytreae*, and suggest that the degree of association between specific insect genetic subgroups and specific plant hosts should be further investigated.

The genetic diversity of African citrus triozids collected from *C. anisata* was particularly striking, as this plant hosted the highest number of haplotypes, including the haplotype most frequently found in Kenya on citrus (Hap1), and the three haplotypes that potentially represent cryptic species (Hap8, Hap9 and Hap 10). This result indicates that *C. anisata* is an important reservoir for African citrus triozid diversity, and that future insights into the genetic diversity of the insect could be obtained from the survey of specimens collected from this particular plant host. In South Africa, where *C. anisata* is commonly infected with LafCl, the bacterium has not been detected on citrus, in spite of the two plant species being frequently found in proximity. In contrast, LafCl has been reported from citrus in Uganda and Tanzania^18,19^. This apparent contradiction requires further study, as it can be hypothesized that the *T. erytreae* haplotypes or cryptic species present on *C. anisata* in South Africa differ from those in Uganda and Tanzania, and do not necessarily feed on citrus.

It is also noteworthy that, among the diversity of genetic lineages found in *T. erytreae*, only one lineage (represented by the closely related Branches I and II) seems to be successful on citrus in Kenya, Tanzania, South Africa and Europe. Interestingly, the haplotype found in Uganda and Ethiopia (Hap7, citrus) represents a distinct branch not found in Kenya or South Africa. This suggests that triozids affecting citrus plants in Ethiopia and Uganda may represent local phylogeographic lineages. Another interesting aspect is the pattern of divergence of Hap8 (Kenya, *C. anisata*) relatively to the other haplotypes. Hap8 was genetically distinct from all other haplotypes with p-distances higher than 4.89%, except for Hap6 (3.63%), which was also found in Kenya on *C. anisata*, and Hap7 (2.82%), the haplotype found in Ethiopia and Uganda. Although the use of p-distances based on DNA barcodes for species delimitation is controversial, and universal threshold cannot be established^30^, this result warrants further investigation for elucidating the taxonomic status of African citrus triozids. For that purpose, phylogenetic, genetic diversity and morphological analyses should be performed using a larger sequence dataset across the geographical range and host diversity of *T. erytreae*. Due to the limitations of mitochondrial DNA for the recovery of deeper divergences, and detection of hybridization and introgression events, definite answers may require the analysis of nuclear sequence data. In addition, the evidence for cryptic diversity in African citrus triozids, currently classified as *T. erytreae*, opens questions regarding potential association between specific genetic lineages of the vector and the genetic diversity of Liberibacter.

Finally, elucidating the phylogeographic structure and genetic diversity of *T. erytreae* can contribute to understand the dynamics of transcontinental dispersal of the insect and associated citrus pathogens. This aspect is critical for the management of triozid-mediated citrus disease in the current scenario of global trade, and the recent evidence for the high potential for invasion of African citrus triozids. For example, China was excluded as the probable source of *D. citri* in California, based on the close relationship of the Californian mitochondrial haplotypes, where the psyllid was detected in 2008, with those found in Florida, where the psyllid was detected in 1998^31^. The characterization of the phylogeography of *T. erytreae* will contribute to elucidate the origin of the recent invasion of Europe, and to model potential routes for global dissemination of citrus diseases transmitted by African citrus triozids.

## Methods

### Sample collection and DNA extraction

Adult triozids were collected during field surveys on citrus orchards and backyard gardens in Ethiopia, Uganda and South Africa (Table S6). Identification of the specimens followed standard of the European and Mediterranean Plant Protection Organization for the identification of *T. erytreae*^32^. Specimens were aspirated from the trees and stored in 96% ethanol until DNA extraction. Geographical location of the sampling sites were recorded using a Garmin eTrex20 instrument (Garmin, USA). One adult specimen per country was randomly chosen for next-generation sequencing (NGS). Total DNA was individually extracted from each adult specimen using the Isolate II Genomic DNA Kit (Bioline, UK), and extracts were stored at −20 °C until further analyses.

### Mitogenome sequencing, assembly and annotation

Total DNA from each of the three specimens was sequenced separately using the Ion Torrent Proton platform (Thermo Fisher Scientific, USA) available at the Central Analytical Facilities of Stellenbosch University, South Africa. Sequence libraries were prepared using the NEXTflex DNA Sequencing Kit for Ion Platforms (PerkinElmer, USA), according to the BI00 Scientific v15.12 protocol. Libraries were pooled and sequenced using the Ion PI HiQ Sequencing Solutions Kit (Life Technologies, USA). The *de novo* assembly of each mitogenome was performed using SPAdes v.3.13.0^33^, and the resulting contigs were identified by BLAST+^34^. Each mitogenome was also mapped and assembled using NC_038142.1 as reference sequence, after excision of a non-annotated 1,689 bp region between ND2 and tRNA^Trp^, using Geneious Prime v2019.1 (https://www.geneious.com)^35^.

Open reading frames of PCGs in both sets of mitogenomes (*de novo* and reference-based assembly) were identified using Geneious Prime, with the invertebrate mitochondrial genetic code. Transfer RNAs (tRNAs) were predicted with ARWEN software (http://130.235.244.92/ARWEN/)^36^, using the default composite metazoan mitochondrial code. Ribosomal RNAs (rRNAs) were estimated by BLASTn search on NCBI (https://blast.ncbi.nlm.nih.gov). Overlapping regions and intergenic spacers were counted manually. Nucleotide composition and AT- and GC-skews were calculated using Geneious Prime, as AT-skew = (A − T)/(A + T) and GC-skew = (G − C)/(G + C).

### Phylogeographic structure and genetic diversity

In order to obtain further insights into the phylogeographic structure of *T. erytreae*, all *COI* sequences publicly available on Genbank as of October 2019 were compiled (Table S4)^25,26^. Multiple sequence alignment was performed using the MAFFT algorithm available on Geneious Prime. The final sequence alignment (n = 89; 571 bp) was used to construct a median-joining network using Network 10 software (http://www.fluxus-engineering.com/sharenet.htm), under the default settings^37^. Genetic divergence among the haplogroups displayed in the network were calculated as pairwise distances (p-distances) under the Kimura 2-parameter model (K2P)^38^, using MEGA X^39^. A neighbour-joining (NJ) tree was also constructed as an alternative display of the relationships among the sequences, using one sequence representative for each geographical region and each host, when the information was available. The NJ tree was constructed using MEGA X based on p-distances under the K2P model, with 1,000 bootstrap replicates. *COI* sequences for 30 other *Trioza* species represented by at least two individuals were retrieved from Genbank for the assessment of intra-specific p-distances within the genus, as described above for the p-distances within *T. erytreae*.

### Phylogenetic analyses

Phylogenetic relationships among Triozidae were reconstructed using the new mitogenomes generated in this study, along with the nine complete mitogenomes available on Genbank for the family as of October 2019, with *Aphis gossypii* and *Schizaphis graminum* (Aphididae) as outgroups (Table S1). The phylogenetic analysis was based on the complement of the 13 PCGs, excluding stop codons. PCG sequences were extracted based on annotations, and concatenated using Geneious Prime. The concatenated sequences were aligned using the MAFFT algorithm^40^ available on Geneious Prime. The best-fitting evolutionary model (GTR + I + G) for the construction of the maximum-likelihood (ML) tree was selected using jModelTest2^41^, according to the AIC and BIC criteria. The ML tree was run using the PhyML 3.1/3.0 aLRT algorithm^42^ available at https://www.phylogeny.fr/. Nodal support was based on 1,000 bootstrap replicates.

## Supplementary information

Supplementary Information.

## Data Availability

The sequences generated in this study were deposited in Genbank under the accession numbers MT416549, MT416550 and MT416551.
