# Author Correction: Mitochondrial genetic variation reveals phylogeographic structure and cryptic diversity in *Trioza erytreae*

**DOI:** 10.1038/s41598-020-76731-w

**Published:** 2020-11-13

**Authors:** Inusa Ajene, Fathiya M. Khamis, Gerhard Pietersen, Barbara van Asch

**Affiliations:** 1 Department of Genetics, Stellenbosch University, Private Bag X1, Matieland, 7602 South Africa; 2 Department of Crop Protection, Ahmadu Bello University, Samaru, 810001 Zaria Nigeria; 3 International Center of Insect Physiology and Ecology, P.O. Box 30772, Nairobi, Kenya

Correction to: *Scientific Reports* 10.1038/s41598-020-65880-7, published online 1 June 2020

Fathiya M. Khamis was omitted from the author list in the original version of this Article. This has been corrected in the PDF and HTML versions of the Article, and in the accompanying Supplementary Information file.

The Author Contributions section now reads:

“B.V.A. designed the study, and I.A., F.K. and G.P. performed the analyses. All authors discussed the results and contributed to the writing of the final version of the manuscript.”

The Acknowledgements statement was also incomplete:

“IA was supported by a German Academic Exchange Service (DAAD) In-Region Postgraduate Scholarship, and a study fellowship from Ahmadu Bello University, Nigeria.”

now reads:

“Inusa Ajene was supported by a German Academic Exchange Service (DAAD) In-Region Postgraduate Scholarship and the German Ministry for Economic Cooperation and Development (BMZ) through GIZ to the project ‘Strengthening Citrus Production Systems through the Introduction of Integrated Pest Management (IPM) Measures for Pests and Diseases in Kenya and Tanzania (SCIPM)’. We would also like to acknowledge icipe core funding provided by the UK Aid from the UK Government, the Swedish International Development Cooperation Agency (Sida), the Swiss Agency for Development and Cooperation (SDC), the Federal Democratic Republic of Ethiopia, and the Kenyan Government.”

