# Supplementary material for: Mitochondrial genetic variation reveals phylogeographic structure and cryptic diversity in Trioza erytreae
Source: Sci Rep. 2020 Jun 1;10:8893. doi: 10.1038/s41598-020-65880-7 (PMC7264336; doi:10.1038/s41598-020-65880-7)
Supplement: Supplementary file 1 — Supplementary Information. [file 41598_2020_65880_MOESM1_ESM.pdf]

Mitochondrial genetic variation reveals phylogeographic structure and cryptic diversity in *Trioza erytreae*

Inusa Ajene[1,2,3], Gerhard Pietersen[1], Barbara van Asch[1]

[1]Department of Genetics, Stellenbosch University, Private Bag X1, Matieland 7602, South Africa

[2]Department of Crop Protection, Ahmadu Bello University, Samaru 810001, Zaria, Nigeria

[3]International Center of Insect Physiology and Ecology, P.O. Box 30772 Nairobi

Supplementary tables

Table S1. List of the 13 complete mitochondrial sequences used in the phylogenetic reconstruction of the family Triozidae (Hemiptera) with common name, broad geographic distribution, GenBank accession number and bibliographic reference. *Aphis gossypii* and *Schizaphis graminum* (Hemiptera: Aphididae) were used as outgroups.

Species	Family	Distribution	Common name	GenBank	Reference	Size (bp)
Aacanthocnema dobsoni	Triozidae	Australia	-	NC_038132.1	Percy et al 2018	15,179
Bactericera cockerelli	Triozidae	North America, Central America, Oceania	Potato psyllid	NC_030055	Wu et al 2016	15,220
Leptynoptera sulfureae	Triozidae	Cocos (Keeling Islands, Indian Ocean Islands	-	NC_038136.1	Percy et al 2018	14,825
Paratrioza sinica	Triozidae	Asia	-	NC_024577	Zhang et al 2014	14,863
Pariaconus pele	Triozidae	Hawaiian islands	-	NC_038138.1	Percy et al 2018	15,080
Trioza anthrisci	Triozidae	Iceland, Nordic countries	-	NC_038141.1	Percy et al 2018	14,918
Trioza erytreae (Uganda)	Triozidae	Africa, Asia, Europe	African citrus triozid	MT416549	This study	15,095
Trioza erytreae (South Africa)	Triozidae	Africa, Asia, Europe	African citrus triozid	MT416550	This study	15,098
Trioza erytreae (Ethiopia)	Triozidae	Africa, Asia, Europe	African citrus triozid	MT416551	This study	15,095
Trioza remota	Triozidae	Europe	-	NC_038143.1	Percy et al 2018	14,950
Trioza urticae	Triozidae	Europe	-	NC_038113.1	Percy et al 2018	15,185
Aphis gossypii	Aphididae	Asia, Africa, North America, Central America, South	Cotton aphid	NC_024581	Zhang et al 2014	15,869
Schizaphis graminum	Aphididae	Asia, Africa, North America, Central America, South	Wheat aphid	NC_006158	Thao et al 2004	15,721

Table S2. Nucleotide composition of the complete mitochondrial sequence of three *Trioza erytreae* specimens collected in Ethiopia, Uganda, and South Africa. AT-skew = (A - T)/(A + T); CG-skew = (G - C)/(G + C).

***Trioza erytreae* Ethiopia**										
**Region**	**A%**	**C%**	**G%**	**T%**	**A+T%**	**G+C%**	**AT-skew**	**GC-skew**	**bp**	**% (size)**
COI	32.8	12.1	12.3	42.8	75.6	24.4	-0.13	0.01	1533	10.16
COII	36.6	14.0	9.8	39.6	76.2	23.8	-0.04	-0.18	664	4.40
ATP8	47.1	10.5	3.9	38.6	85.7	14.4	0.10	-0.46	153	1.01
ATP6	38.4	12.0	7.7	41.9	80.3	19.7	-0.04	-0.22	675	4.47
COIII	36.5	11.1	9.2	43.2	79.7	20.3	-0.08	-0.09	783	5.19
ND3	40.5	9.7	7.7	42.2	82.7	17.4	-0.02	-0.11	351	2.33
ND5	45.4	11.8	8.6	34.2	79.6	20.4	0.14	-0.16	1618	10.72
ND4	45.5	13.3	7.5	33.8	79.3	20.8	0.15	-0.28	1245	8.25
ND4L	51.4	10.8	5.9	31.9	83.3	16.7	0.23	-0.29	288	1.91
ND6	37.5	9.6	6.7	46.3	83.8	16.3	-0.11	-0.18	480	3.18
CYTB	33.9	12.4	9.6	44.0	77.9	22.0	-0.13	-0.13	1143	7.57
ND1	49.3	12.3	8.5	29.8	79.1	20.8	0.25	-0.18	918	6.08
16s rRNA	41.1	13.9	6.2	38.8	79.9	20.1	0.03	-0.38	1146	7.59
12s rRNA	39.3	12.8	7.9	40.0	79.3	20.7	-0.01	-0.24	685	4.54
ND2	37.6	10.5	6.5	45.4	83.0	17.0	-0.09	-0.24	969	6.42
PCGs	40.1	11.9	8.7	39.3	79.4	20.6	0.01	-0.16	10820	71.68
tRNAs	41.5	11.6	9.4	37.5	79.0	21.0	0.05	-0.10	1365	9.04
rRNAs	40.4	13.5	6.8	39.3	79.7	20.3	0.01	-0.33	1831	12.13
AT-rich region	44.7	6.1	5.1	44.0	88.7	11.2	0.01	-0.09	993	6.58
Complete mtDNA	40.6	11.7	8.3	39.4	80.0	20.0	0.02	-0.17	15095	100.00

***Trioza erytreae* South Africa**										
**Region**	**A%**	**C%**	**G%**	**T%**	**A+T%**	**G+C%**	**AT-skew**	**GC-skew**	**bp**	**% (size)**
COI	32.9	12.2	12.4	42.5	75.4	24.6	-0.13	0.01	1533	10.16
COII	37.2	13.1	9.8	39.8	77.0	22.9	-0.03	-0.14	664	4.40
ATP8	48.4	9.2	2.0	40.5	88.9	11.2	0.09	-0.64	153	1.01
ATP6	38.5	12.0	7.6	41.9	80.4	19.6	-0.04	-0.22	675	4.47
COIII	36.4	10.9	9.3	43.4	79.8	20.2	-0.09	-0.08	783	5.19
ND3	40.5	9.1	8.0	42.5	83.0	17.1	-0.02	-0.06	351	2.33
ND5	45.4	11.4	8.3	34.8	80.2	19.7	0.13	-0.16	1618	10.72
ND4	45.4	13.2	7.6	33.8	79.2	20.8	0.15	-0.27	1245	8.25
ND4L	52.1	11.5	5.6	30.9	83.0	17.1	0.26	-0.35	288	1.91
ND6	37.1	9.8	6.9	46.3	83.4	16.7	-0.11	-0.17	480	3.18
CYTB	33.5	13.0	10.0	43.4	76.9	23.0	-0.13	-0.13	1143	7.57
ND1	49.3	12.5	8.3	29.9	79.2	20.8	0.24	-0.20	923	6.11
16s rRNA	41.0	13.7	6.1	39.2	80.2	19.8	0.02	-0.38	1148	7.61
12s rRNA	39.4	12.8	7.7	40.0	79.4	20.5	-0.01	-0.25	685	4.54
ND2	38.0	10.6	6.1	45.3	83.3	16.7	-0.09	-0.27	969	6.42
PCGs	40.1	11.8	8.7	39.3	79.4	20.5	0.01	-0.15	10825	71.71
tRNAs	41.3	11.4	9.5	37.9	79.2	20.9	0.04	-0.09	1365	9.04
rRNAs	40.4	13.4	6.7	39.5	79.9	20.1	0.01	-0.33	1831	12.13
AT-rich region	44.6	5.8	5.8	43.8	88.4	11.6	0.01	0.00	993	6.58
Complete mtDNA	40.6	11.6	8.3	39.5	80.1	19.9	0.01	-0.17	15095	100.00

***Trioza erytreae* Uganda**										
**Region**	**A%**	**C%**	**G%**	**T%**	**A+T%**	**G+C%**	**AT-skew**	**GC-skew**	**bp**	**% (size)**
COI	32.8	12.1	12.3	42.8	75.6	24.4	-0.13	0.01	1533	10.16
COII	36.4	14.0	9.9	39.6	76.0	23.9	-0.04	-0.17	664	4.40
ATP8	47.1	10.5	3.9	38.6	85.7	14.4	0.10	-0.46	153	1.01
ATP6	38.7	12.0	7.4	41.9	80.6	19.4	-0.04	-0.24	675	4.47
COIII	36.5	11.0	9.2	43.2	79.7	20.2	-0.08	-0.09	783	5.19
ND3	40.5	9.7	7.7	42.2	82.7	17.4	-0.02	-0.11	351	2.33
ND5	45.4	11.8	8.7	34.2	79.6	20.5	0.14	-0.15	1618	10.72
ND4	45.5	13.3	7.5	33.7	79.2	20.8	0.15	-0.28	1245	8.25
ND4L	51.1	10.6	6.4	31.9	83.0	17.0	0.23	-0.25	288	1.91
ND6	37.5	9.6	6.7	46.3	83.8	16.3	-0.11	-0.18	480	3.18
CYTB	33.9	12.5	9.6	43.9	77.8	22.1	-0.13	-0.13	1143	7.57
ND1	49.0	12.2	8.8	30.0	79.0	21.0	0.24	-0.16	923	6.11
16s rRNA	41.0	13.9	6.3	38.8	79.8	20.2	0.03	-0.38	1146	7.59
12s rRNA	39.3	12.8	7.9	40.0	79.3	20.7	-0.01	-0.24	685	4.54
ND2	37.4	10.6	6.6	45.4	82.8	17.2	-0.10	-0.23	965	6.39
PCGs	40.0	11.9	8.8	39.3	79.3	20.7	0.01	-0.15	10821	71.69
tRNAs	41.4	11.6	9.5	37.5	78.9	21.1	0.05	-0.10	1365	9.04
rRNAs	40.4	13.5	6.9	39.3	79.7	20.4	0.01	-0.32	1831	12.13
AT-rich region	44.6	6.1	5.1	44.0	88.6	11.2	0.01	-0.09	993	6.58
Complete mtDNA	40.5	11.7	8.4	39.4	79.9	20.1	0.01	-0.16	15095	100.00

Table S3. Pairwise comparison of three *Trioza erytreae* specimens collected in Ethiopia (TE-ETH), Uganda (TE-UG), and South Africa (TE-SA) given as the number of single nucleotide polymorphisms (SNPs) and non-synonymous amino acid substitutions (NS) in the complete complement of 13 mitochondrial protein coding genes.

		TE-UG vs TE-SA				TE-ETH vs SA-TE				TE-ETH vs TE-UG			
**Gene**	**Length (bp)**	**SNPs**	**%**	**NS**	**%**	**SNPs**	**%**	**NS**	**%**	**SNPs**	**%**	**NS**	**%**
ATP6	674	15	1.76	6	0.89	15	1.76	5	0.74	2	0.21	1	0.15
ATP8	153	7	2.56	3	1.96	7	2.56	3	1.96	0	0.00	0	0.00
COI	1533	28	2.80	0	0.00	30	2.71	0	0.00	2	0.09	0	0.00
COII	664	20	2.94	3	0.45	19	2.83	3	0.45	1	0.33	0	0.00
COIII	783	29	2.81	10	1.28	28	2.73	9	1.15	3	0.00	1	0.13
CYTB	1143	32	3.58	5	0.44	31	3.52	5	0.44	1	0.12	0	0.00
ND1	918	27	1.83	6	0.65	26	1.96	7	0.76	3	0.13	1	0.11
ND2	968	17	3.01	5	0.52	17	2.86	6	0.62	2	0.15	1	0.10
ND3	351	9	2.71	3	0.85	9	2.71	3	0.85	0	0.00	0	0.00
ND4	1245	35	4.58	4	0.32	34	4.58	4	0.32	0	0.00	0	0.00
ND4L	288	1	2.08	6	2.08	1	2.08	0	0.00	0	0.00	0	0.00
ND5	1618	58	3.70	16	0.99	57	3.58	16	0.99	2	0.38	0	0.00
ND6	480	13	2.23	5	1.04	13	2.23	6	1.25	0	0.30	0	0.00
Total		291		72		287		67		16		4	

Table S4. List of publicly available and new cytochrome c subunit 1 (*COI*) sequences (n = 89) reported as *Trioza erytreae* (Hemiptera: Triozidae), and used for the construction of median-joining network, neighbor-joining tree, and estimates of genetic distances and genetic diversity.

**Country**	**Name**	**Accession**	**REF**	**Haplotype**
Ethiopia	TE-ETH	Upon acceptance	This study	Hap 7
Kenya	Kenya *citrus* 01	KY754595.1	Khamis et al 2017	Hap 1
Kenya	Kenya *citrus* 02	KY754596.1	Khamis et al 2017	Hap 1
Kenya	Kenya *citrus* 03	KY754597.1	Khamis et al 2017	Hap 1
Kenya	Kenya *citrus* 04	KY754599.1	Khamis et al 2017	Hap 1
Kenya	Kenya *citrus* 05	KY754600.1	Khamis et al 2017	Hap 1
Kenya	Kenya *citrus* 06	KY754610.1	Khamis et al 2017	Hap 1
Kenya	Kenya *citrus* 07	KY754613.1	Khamis et al 2017	Hap 1
Kenya	Kenya *citrus* 08	KY754614.1	Khamis et al 2017	Hap 1
Kenya	Kenya *citrus* 09	KY754615.1	Khamis et al 2017	Hap 1
Kenya	Kenya *citrus* 10	KY754619.1	Khamis et al 2017	Hap 1
Kenya	Kenya *citrus* 11	KY754620.1	Khamis et al 2017	Hap 1
Kenya	Kenya *citrus* 12	KY754621.1	Khamis et al 2017	Hap 1
Kenya	Kenya *citrus* 13	KY754622.1	Khamis et al 2017	Hap 1
Kenya	Kenya *citrus* 14	KY754623.1	Khamis et al 2017	Hap 1
Kenya	Kenya *citrus* 15	KY754624.1	Khamis et al 2017	Hap 1
Kenya	Kenya *citrus* 16	KY754625.1	Khamis et al 2017	Hap 1
Kenya	Kenya *citrus* 17	KY754626.1	Khamis et al 2017	Hap 1
Kenya	Kenya *citrus* 18	KY754627.1	Khamis et al 2017	Hap 1
Kenya	Kenya *citrus* 19	KY754628.1	Khamis et al 2017	Hap 1
Kenya	Kenya *citrus* 20	KY754629.1	Khamis et al 2017	Hap 1
Kenya	Kenya *citrus* 21	KY754630.1	Khamis et al 2017	Hap 1
Kenya	Kenya *citrus* 22	KY754631.1	Khamis et al 2017	Hap 1
Kenya	Kenya *citrus* 23	KY754632.1	Khamis et al 2017	Hap 1
Kenya	Kenya *citrus* 24	KY754633.1	Khamis et al 2017	Hap 1
Kenya	Kenya *citrus* 25	KY754634.1	Khamis et al 2017	Hap 1
Kenya	Kenya *citrus* 26	KY754635.1	Khamis et al 2017	Hap 1
Kenya	Kenya *citrus* 27	KY754641.1	Khamis et al 2017	Hap 1
Kenya	Kenya *citrus* 28	KY754642.1	Khamis et al 2017	Hap 1
Kenya	Kenya *citrus* 29	KY754643.1	Khamis et al 2017	Hap 1
Kenya	Kenya *citrus* 30	KY754644.1	Khamis et al 2017	Hap 1
Kenya	Kenya *citrus* 31	KY754645.1	Khamis et al 2017	Hap 1
Kenya	Kenya *citrus* 32	KY754646.1	Khamis et al 2017	Hap 1
Kenya	Kenya *citrus* 33	KY754647.1	Khamis et al 2017	Hap 1
Kenya	Kenya *citrus* 34	KY754648.1	Khamis et al 2017	Hap 1
Kenya	Kenya *citrus* 35	KY754649.1	Khamis et al 2017	Hap 1
Kenya	Kenya *citrus* 36	KY754650.1	Khamis et al 2017	Hap 1
Kenya	Kenya *citrus* 37	KY754651.1	Khamis et al 2017	Hap 1
Kenya	Kenya *citrus* 38	KY754652.1	Khamis et al 2017	Hap 1
Kenya	Kenya *citrus* 39	KY754653.1	Khamis et al 2017	Hap 1
Kenya	Kenya *citrus* 40	KY754654.1	Khamis et al 2017	Hap 1
Kenya	Kenya *citrus* 41	KY754655.1	Khamis et al 2017	Hap 1
Kenya	Kenya *citrus* 42	KY754656.1	Khamis et al 2017	Hap 1
Kenya	Kenya *citrus* 43	KY754611.1	Khamis et al 2017	Hap 1
Kenya	Kenya *citrus* 44	KY754612.1	Khamis et al 2017	Hap 2
Kenya	Kenya *citrus* 45	KY754598.1	Khamis et al 2017	Hap 3
Kenya	Kenya *Clausena anisata* 1	KY754616.1	Khamis et al 2017	Hap 1
Kenya	Kenya *Clausena anisata* 2	KY754617.1	Khamis et al 2017	Hap 1
Kenya	Kenya *Clausena anisata* 3	KY754618.1	Khamis et al 2017	Hap 1
Kenya	Kenya *Clausena anisata* 4	KY754586.1	Khamis et al 2017	Hap 6
Kenya	Kenya *Clausena anisata* 5	KY754585.1	Khamis et al 2017	Hap 8
Kenya	Kenya *Clausena anisata* 6	KY754584.1	Khamis et al 2017	Hap 9
Kenya	Kenya *Clausena anisata* 7	KY754587.1	Khamis et al 2017	Hap 10
Kenya	Kenya *Murraya koenigii* 1	KY754601.1	Khamis et al 2017	Hap 1
Kenya	Kenya *Murraya koenigii* 2	KY754602.1	Khamis et al 2017	Hap 1
Kenya	Kenya *Murraya koenigii* 3	KY754603.1	Khamis et al 2017	Hap 1
Kenya	Kenya *Murraya koenigii* 4	KY754604.1	Khamis et al 2017	Hap 1
Kenya	Kenya *Murraya koenigii* 5	KY754605.1	Khamis et al 2017	Hap 1
Kenya	Kenya *Murraya koenigii* 6	KY754606.1	Khamis et al 2017	Hap 1
Kenya	Kenya *Murraya koenigii* 7	KY754607.1	Khamis et al 2017	Hap 1
Kenya	Kenya *Murraya koenigii* 8	KY754608.1	Khamis et al 2017	Hap 1
Kenya	Kenya *Murraya koenigii* 9	KY754609.1	Khamis et al 2017	Hap 1
Kenya	Kenya *Stephania abyssinica* 1	KY754636.1	Khamis et al 2017	Hap 1
Kenya	Kenya *Stephania abyssinica* 2	KY754637.1	Khamis et al 2017	Hap 1
Kenya	Kenya *Stephania abyssinica* 3	KY754638.1	Khamis et al 2017	Hap 1
Kenya	Kenya *Stephania abyssinica* 4	KY754639.1	Khamis et al 2017	Hap 1
Kenya	Kenya *Stephania abyssinica* 5	KY754640.1	Khamis et al 2017	Hap 1
Portugal	Portugal Madeira	MK285558.1	Pérez-Rodríguez et al 2019	Hap 4
South Africa	SA *citrus* 1	KY754589.1	Khamis et al 2017	Hap 3
South Africa	SA *citrus* 2	KY754591.1	Khamis et al 2017	Hap 3
South Africa	SA *citrus* 3	KY754592.1	Khamis et al 2017	Hap 3
South Africa	SA *citrus* 4	KY754593.1	Khamis et al 2017	Hap 3
South Africa	SA *citrus* 5	KY754590.1	Khamis et al 2017	Hap 4
South Africa	SA *citrus* 6	KY754594.1	Khamis et al 2017	Hap 4
South Africa	SA Nelspruit 1	MK285554.1	Pérez-Rodríguez et al 2019	Hap 5
South Africa	SA Nelspruit 2	MK285555.1	Pérez-Rodríguez et al 2019	Hap 5
South Africa	SA Pretoria 1	MK285559.1	Pérez-Rodríguez et al 2019	Hap 4
South Africa	SA Pretoria 2	MK285560.1	Pérez-Rodríguez et al 2019	Hap 5
South Africa	SA Tzaneen 1	MK285556.1	Pérez-Rodríguez et al 2019	Hap 3
South Africa	SA Tzaneen 2	MK285557.1	Pérez-Rodríguez et al 2019	Hap 5
South Africa	TE-SA	Upon acceptance	This study	Hap 5
Spain	Spain Galicia Aldan	MK285548.1	Pérez-Rodríguez et al 2019	Hap 4
Spain	Spain Galicia Areeiro	MK285549.1	Pérez-Rodríguez et al 2019	Hap 4
Spain	Spain Galicia Portonovo	MK285550.1	Pérez-Rodríguez et al 2019	Hap 4
Spain	Spain Gran Canaria	MK285553.1	Pérez-Rodríguez et al 2019	Hap 4
Spain	Spain Tenerife 1	MK285551.1	Pérez-Rodríguez et al 2019	Hap 4
Spain	Spain Tenerife 2	MK285552.1	Pérez-Rodríguez et al 2019	Hap 4
Tanzania	Tanzania *citrus*	KY754588.1	Khamis et al 2017	Hap 1
Uganda	TE-UG	Upon acceptance	This study	Hap 7

Table S5. Estimates of minimum, maximum and average intra-specific genetic divergence in 31 species belonging to the genus *Trioza* (Hemiptera: Triozidae), calculated as percentage of pairwise distances under the Kimura 2-parameter model.

Species	n	bp	Min	Max	Mean	SE
Trioza acuta	4	395	0.00	1.54	0.80	0.300
Trioza anceps	2	472	14.27	14.27	14.30	1.900
Trioza apicalis	3	430	0.00	0.23	0.20	0.200
Trioza aylmeriae	5	355	0.00	0.00	0.00	0.000
Trioza barrettae	3	400	0.25	1.52	1.00	0.400
Trioza bifida	3	395	0.00	1.02	0.50	0.200
Trioza brevigenae	9	658	0.00	0.00	0.00	0.000
Trioza colorata	4	395	0.00	1.02	0.50	0.200
Trioza compressa	4	395	0.00	0.51	0.30	0.200
Trioza curta	2	395	0.00	0.00	0.00	0.000
Trioza dacrydii	2	395	0.51	0.51	0.50	0.300
Trioza decurvata	3	395	2.06	3.92	3.10	0.700
Trioza discariae	4	395	0.00	0.25	0.10	0.100
Trioza doryphora	4	395	0.00	0.00	0.00	0.000
Trioza erytreae	52	535	0.00	11.71	0.90	0.200
Trioza eugeniae	7	395	0.00	2.33	0.90	0.300
Trioza falcata	4	395	0.00	1.28	0.90	0.400
Trioza fasciata	2	395	3.93	3.93	3.90	1.000
Trioza gourlayi	2	395	0.25	0.25	0.30	0.200
Trioza grallata	2	470	0.00	0.00	0.00	0.000
Trioza hebicola	2	395	0.25	0.25	0.30	0.200
Trioza incrustata	2	470	0.00	0.00	0.00	0.000
Trioza irregularis	11	395	0.00	1.28	0.60	0.200
Trioza obscura	3	395	0.00	1.54	1.00	0.400
Trioza obunca	2	472	0.00	0.00	0.00	0.000
Trioza panacis	4	395	0.00	0.51	0.30	0.200
Trioza remota	2	472	0.43	0.43	0.40	0.300
Trioza subacuta	5	395	0.00	0.51	0.40	0.200
Trioza subvexa	5	395	0.25	3.39	2.00	0.600
Trioza tricornuta	6	370	0.00	1.65	1.00	0.400
Trioza urticae	96	472	0.00	4.18	1.60	0.300

Table S6. Sample data of the tree adult specimens of the African citrus triozid *Trioza erytreae* (Hemiptera: Triozidae) collected in Ethiopia (TE-ETH), Uganda (TE-UG) and South Africa (TE-SA) on *Citrus sinensis* (L.) Osbek, and used for next-generation sequencing of the complete mitochondrial genomes.

**Sample**	**Species**	**Collection date**	**Country**	**Latitude**	**Longitude**
TE-ETH	*Trioza erytreae*	19-Nov-17	Ethiopia	11.3917	37.0741
TE-UG	*Trioza erytreae*	24-Mar-17	Uganda	-0.4964	31.5548
TE-SA	*Trioza erytreae*	10-Jul-19	South Africa	-25.4791	30.9928

Supplementary figures

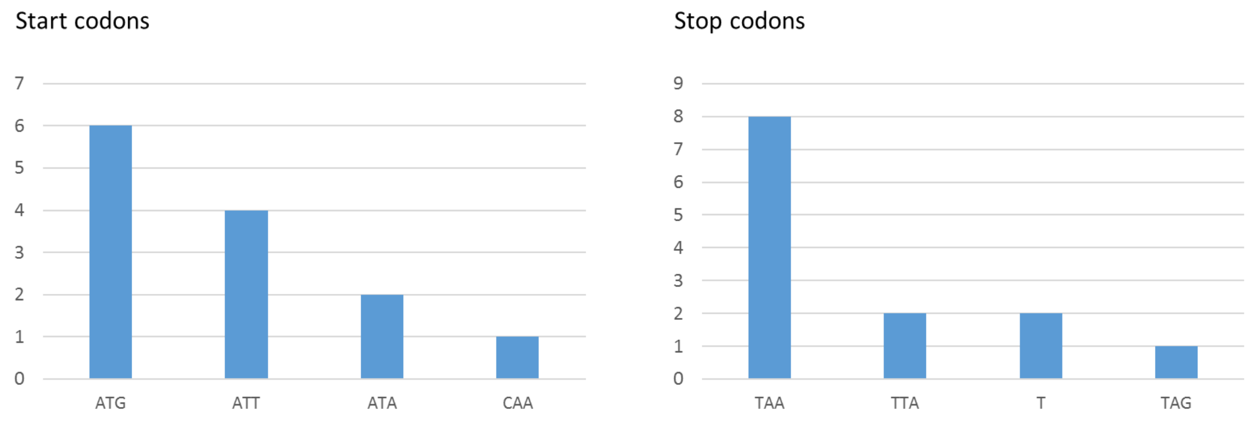

Figure S1. Start and stop codons found in the complete complement of 13 mitochondrial genes of *Trioza erytreae*.
